# Topics of Public Interest

**Published:** 1909-11

**Authors:** 


### TOPICS OF PUBLIC INTEREST.

### Mortality Statistics of Next Year.

REVISED VERSION OF CLASSIFICATION OF CENSUS TO GO INTO EFFECT.

United States Census Director, E. Dana Durand, October 11, 1909, promulgated new rules and instructions for the purpose of securing more complete and accurate transcripts of deaths occurring in the selected death registration states and cities of the United States. These transcripts are obtained every month by the Census Bureau from nearly all of the city and state registrars in the census death registration area, and they form the basis of the mortality statistics prepared by the Division of Vital Statistics, under Chief Statistician, Dr. Cressy L. Wilbur.

This action is expected to result in the presentation of the most scientific and trustworthy mortality statistics ever compiled in connection with a decennial United States Census, which affords the population bases for the 1910 death rates. In addition to this important step toward more reliable data, the new revised version of the classification of the causes of death, as adopted at the Paris conference for the second decennial revision of the International Classification, will go into effect January 1, 1910, in the census registration area. Supplementing these will be the use of the new United States standard death certificates which it is believed the organised registration officials forming the vital statistic section of the American Public Health Association will adopt for the report of deaths commencing January 1, 1910, at the Richmond, Va., meeting, October 19, 22, 1909.

In his communication to the state registrars, Director Durand states that in their work of coöperation it is of the greatest importance that there should be exact agreement between the number of deaths as compiled by the state officers and by the Census Bureau, at least with respect to the total number of deaths reported for each month in each state, county and city. Differences occur at present, which are not creditable to American statistics. For the purpose of preventing such differences, a monthly shipment check list, showing the deaths by months and areas, has been prepared and will be supplied to each state registrar.

He asks transcribers to follow absolutely the instructions for copying and advises tests to ascertain correctness. Permanent transcribers are preferred because of the skill acquired. Local registrars should be compelled to make returns on time. No effective registration can exist when the central office permits tardiness. The credit of the state service must suffer, the director states, from heedless and incompetent work, and the compensation paid for the returns is sufficient to entitle the government to thoroughly reliable transcripts, promptly transmitted, and containing all of the statistical data required to be registered under the state law.

To the city registrars, the director suggests they note the instructions to state registrars. He states that a city registrar should have in his hands the certificate of every death that occurs, with absolutely no exception, before any disposition is made of the body; hence, there should be no occasion for certificates, filed many days after the close of each month or year. The corrections should be obtained before the burial or removal permit is issued. No imperfect certificates or unsatisfactory statements of cause of death should be accepted. When overlooked, however, they may be corrected readily by special blank or telephone, and city returns should therefore be superior in quality and completeness.

In conclusion the director states that with the cordial co-operation of state and city registration officials, the value of the mortality statistics of the United States will be greatly improved. It is especially requested that every effort be made to carry out faithfully the recommendations for the remaining months of the present year, so that the entire returns for the year 1910, which are especially important because of the comparisons possible with the population data of the thirteenth census, may be in complete agreement for all of the states and cities of the United States. Special circulars of instructions will be issued relative to the reporting of occupations and causes of death. It is hoped that the new standard certificate and the approved instructions, may be adopted by all of the registration states and cities, so that thoroughly comparable returns may be instituted for the decade beginning January 1, 1910.

Dr. Wilbur, who was one of the American delegates at the second decennial revision, stated today, October 11, 1909, that the opportunity of starting out with the use of the revised classification for the mortality statistics relating to the actual census year, is of the greatest value. It is highly gratifying, he said, that the wishes of the United States for the advancement of the date of the international revision from 1909 to 1910, were acceded to by the French government and the other countries participating.

In accordance with a resolution of the International Commission, an official version of the revised titles is to be prepared in each language represented. The English translation has been made by Dr. Wilbur, aided by the other American delegates and by Honorable G. W. Knibbs, Commonwealth Statistician of Australia. This will provide precisely the same tabular list for all English-speaking countries that have adopted the international classification.

The active interest of the United States in the promotion of international uniformity was accorded a very graceful recognition in the bestowing of the vice-presidency of the International Commission upon Dr. Wilbur, who was called upon to preside over one of the sessions.

The next revision will be called in 1919, and under the auspices of the French Government, unless other provision is made. Dr. Wilbur said it is to be hoped, however, in view of the great advancement of American vital statistics and the important part this country has played in the extension of the international classification, that the third decennial revision will be called by the American government to meet at Washington.

